# Recent Developments in Surface-Enhanced Raman Spectroscopy and Its Application in Food Analysis: Alcoholic Beverages as an Example

**DOI:** 10.3390/foods11142165

**Published:** 2022-07-21

**Authors:** Lijiao Li, Xiaonian Cao, Ting Zhang, Qian Wu, Peng Xiang, Caihong Shen, Liang Zou, Qiang Li

**Affiliations:** 1 Key Laboratory of Coarse Cereal Processing, Ministry of Agriculture and Rural Affairs, Sichuan Engineering & Technology Research Center of Coarse Cereal Industrialization, School of Food and Biological Engineering, Chengdu University, Chengdu 610106, China; J14736449833@126.com (L.L.); z1377311342@126.com (T.Z.); w2394045772@126.com (Q.W.); xiangpeng110@126.com (P.X.); zouliang@cdu.edu.cn (L.Z.); 2 Luzhou Laojiao Co., Ltd., Luzhou 646000, China; caozengjia@163.com (X.C.); shench@lzlj.com (C.S.); 3 National Engineering Research Center of Solid-State Brewing, Luzhou 646000, China; 4 Postdoctoral Research Station of Luzhou Laojiao Company, Luzhou 646000, China

**Keywords:** surface enhanced Raman spectroscopy, technical advance, food safety, component detection

## Abstract

Surface-enhanced Raman spectroscopy (SERS) is an emerging technology that combines Raman spectroscopy and nanotechnology with great potential. This technology can accurately characterize molecular adsorption behavior and molecular structure. Moreover, it can provide rapid and sensitive detection of molecules and trace substances. In practical application, SERS has the advantages of portability, no need for sample pretreatment, rapid analysis, high sensitivity, and ‘fingerprint’ recognition. Thus, it has great potential in food safety detection. Alcoholic beverages have a long history of production in the world. Currently, a variety of popular products have been developed. With the continuous development of the alcoholic beverage industry, simple, on-site, and sensitive detection methods are necessary. In this paper, the basic principle, development history, and research progress of SERS are summarized. In view of the chemical composition, the beneficial and toxic components of alcoholic beverages and the practical application of SERS in alcoholic beverage analysis are reviewed. The feasibility and future development of SERS are also summarized and prospected. This review provides data and reference for the future development of SERS technology and its application in food analysis.

## 1. Introduction

### 1.1. Basic Principles of Raman Scattering

In the past few decades, vibration spectroscopy has been widely used in various fields of detection and evaluation because of its rapid and non-destructive analysis method, and has become one of the most promising detection technologies [1]. This technology includes infrared spectroscopy and Raman spectroscopy [2]. Both of them belong to the molecular vibration spectrum, with infrared spectrum belonging to the absorption spectrum and Raman spectrum belonging to the scattering spectrum. They have different mechanisms, but complement each other. Raman scattering is caused by the difference in polarizability or shape of electron distribution in molecular vibration, while infrared absorption requires the inherent dipole moment to change with molecular vibration [3]. Therefore, infrared spectroscopy is often used to study the asymmetric vibration of polar groups, and Raman spectroscopy is often used to study the symmetric vibration of non-polar groups and skeletons. Raman spectrum can cover the wave number range of 40–4000 cm^−1^ at the same time, and can be used for the analysis of organic and inorganic substances. In addition, Raman spectroscopy can be used to determine aqueous solutions, while infrared spectroscopy cannot [4]. In conclusion, Raman spectroscopy has great development potential.

**Figure 1:**
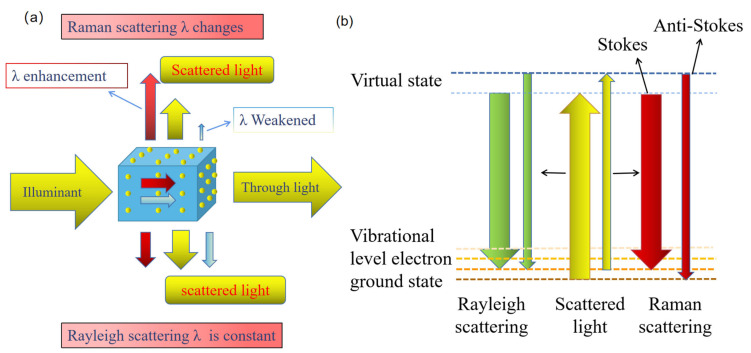
Schematic diagram of the generating principle of Raman scattering. (**a**) When light passes through the object, the energy is divided into penetrating light and reflected light, and the scattered light is divided into Raman scattering and Rayleigh scattering according to the change of energy. (**b**) The Raman and Rayleigh scattering principles in terms of the vibrational energy levels of electrons in objects.

In 1928, Indian physicist Chandrashekhara Venkata Raman discovered a new phenomenon of light scattering, namely inelastic scattering, called Raman spectroscopy, and won the Nobel Prize in physics in 1930 [5]. When light shines on the sample, part of the light directly penetrates the sample with an unchanged direction and unchanged energy. Some of the light is scattered in all directions. The scattered light with changing direction and constant energy is called Rayleigh scattering. A change in the direction of scattered light accompanied by a change in energy (increase or decrease) is called Raman scattering, which includes Stokes lines (energy decreases) and anti-Stokes lines (energy increase) (Figure 1) [6]. The difference between the frequency of the Stokes and anti-Stokes and the frequency of the excited light source is called the Raman shift. The change of vibrational energy level of different molecules leads to the change of Raman shift, thus identifying different molecules [7]. Therefore, Raman scattering can be used to study the vibrational energy level of the target molecule in the detected sample (different molecules of different substances have different vibrational energy levels due to different chemical bonds and groups), so as to analyze the structural composition characteristics of molecules, which is also known as the ‘molecular fingerprint spectrum’ [8].

### 1.2. Raman Spectroscopy Technology and Its Advantages

Compared with fluorescence and infrared spectroscopy, Raman spectroscopy has the following advantages [9]: (1) It has high spectral resolution and narrow bandwidth, which is 10–100 times that of fluorescence scattering of organic dye or quantum dot. This facilitates the simultaneous detection of different analytes, including inorganic and organic matter, in multiple analyses; (2) Raman spectroscopy has low requirements for sample preparation, less contact with samples, no interference from CO_2_ and water, simple measurement, and provides rapid and non-destructive analysis. At the same time, Raman technology has high specificity and sensitivity to various chemical components. It has advantages in the detection of chemical dopants and additives, as well as water quality analysis; (3) The Raman spectrum reflects the molecular characteristics of the sample; (4) Raman spectroscopy has high plasticity and development potential, and a variety of highly sensitive technologies have been innovated based on this technology. SERS is versatile, and active nanosubstrates can be designed with different sizes, shapes, and coatings according to the detection purposes and substances. Therefore, Raman spectroscopy technology is gradually exploited and used as an indispensable detection method [6].

### 1.3. Development and Application Potential of Raman Spectroscopy Technology

Raman spectroscopy is a universal non-destructive technique for fluid inclusion analysis, with applications ranging from the qualitative detection of solid, liquid and gas components to the identification of polyatomic ions in solutions. This technique is commonly used to calculate the density of CO_2_ fluids, the chemical properties of aqueous fluids, and the mole ratio of gaseous mixtures in the form of inclusions [10]. Ordinary Raman spectroscopy has some limitations in analysis and detection, such as: (1) For the detection of low concentration substances, the Raman signal is weak; (2) It is easily affected by optical system parameters; (3) The presence of fluorescence scattering can affect the signal of the sample. Therefore, the Raman spectrum is mostly used to detect solid samples or aqueous solution of high concentration, but is not suitable for trace component analysis [11]. In view of the limitations of Raman spectroscopy, scientists continue to explore and innovate Raman spectroscopy technology [12]. In recent years, the following advancements have been reported: (1) It is found that metallic or non-metallic nanomaterials and nanostructures can be used as active substrates for Raman spectroscopy to enhance the Raman signal of tested samples; (2) Advanced instruments and methods, including Raman microscopy, cutting-edge enhanced Raman, transverse microradiography, extraction instruments, and stoichiometry methods, have improved the reliability of Raman spectroscopy detection [13]; (3) The effectiveness of Raman in biomedical imaging and disease diagnosis [14]; (4) The design and control of ultrafast lasers using nonlinear Raman processes; (5) The application of Raman spectroscopy in art conservation [14], archaeology [15], geology [16], and other emerging fields.

### 1.4. Development History and Principle of Surface-Enhanced Raman Spectroscopy (SERS)

In 1973, Martin Fleischmann, Patrick J. Hendra, and A. James McQuillan discovered pyridine adsorbed on electrochemically rough silver had a strong Raman spectral signal. Based on their observations, surface-enhanced Raman scattering was first discovered [17]. In 1974, they observed a significant increase in the surface Raman signal when pyridine molecules adhered to a silver electrode, which is approximately six orders of magnitude higher than that without silver electrode [18]. They attributed the signal enhancement to the rough surface area. This finding has been verified in recent years. In 1978, Moskovits first explained the effect of surface plasmons on SERS enhancement of rough silver electrodes and predicted the possibility of occurrence on the surface of silver (Ag) and copper (Cu) colloids [19]. In 1979, Creighton et al. successfully tested this prediction using gold and silver colloids [20]. In summary, the findings and conclusions of these researchers provide a clear direction for the development of SERS based on plasmonic nanostructures.

**Figure 2:**
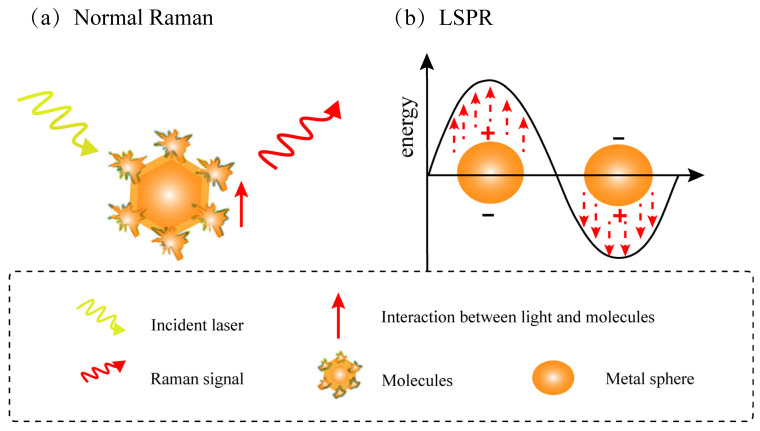
Source of Raman spectral signal. (**a**) Signal generated by light interaction with molecules. (**b**) LSPR (local surface plasmon resonance) signals generated by interactions between metal particles.

**Figure 3:**
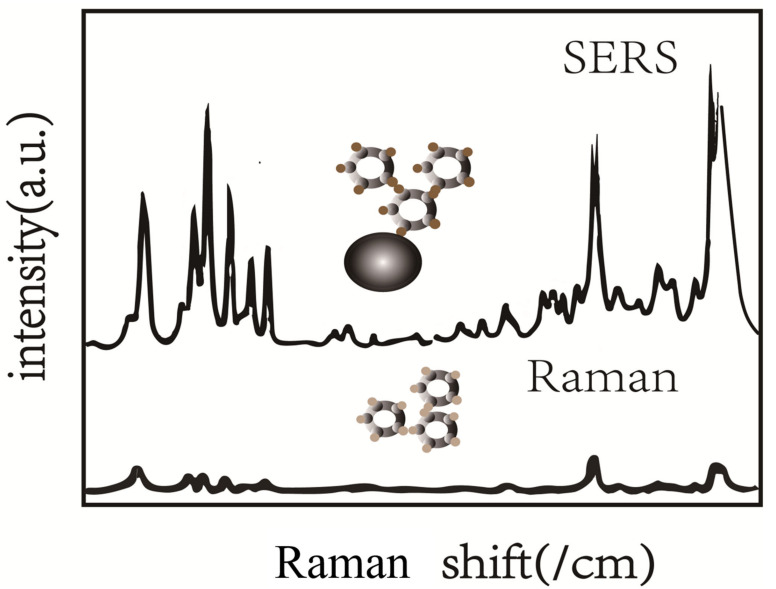
SERS and ordinary Raman spectroscopy. Compared with ordinary Raman, SERS can generate a more intense Raman signal, and the enhancement coefficient can be as high as 10^10^–10^11^.

Surface-enhanced Raman scattering is a sensitive technology that amplifies Raman intensity by using plasmon effect. The enhancement of Raman spectral intensity mainly comes from the electromagnetic interaction between light and metal, which greatly enhances the surrounding electromagnetic field through plasmon resonance excitation (hotspot) [21]. The Raman signal is amplified through the combination of the specific scattering of the target molecule and the excitation of local surface plasmons of the surface material, which combines the phenomena of light–molecular interactions (vibrational spectroscopy) (Figure 2a) and light–metal interactions (local surface plasmon resonance) (Figure 2b). According to a recent report, the enhancement factor of the metal surface can be up to 10^6^–10^8^ [22]. Research has also found that the largest enhancement occurs on rough surfaces at the nanoscale, and the enhancement factor can be as high as 10^10^–10^11^ [23]. The advantages of SERS are described in Figure 3 [11]. It has been found that the higher the surface roughness of nanoparticles, the better the enhancement effect [24].

Many different theories have been proposed to explain why some roughened metal surfaces achieve Raman enhancement. Raman scattering of molecules is the polarization of molecules under the action of external electric field. In the process of re-emission, the alternating polarization of molecules receives the modulation of interatomic vibration, thus generating Raman scattering light. The enhancement of scattered light may be due to the enhancement of the local electric field acting on the molecule and the change of the molecular polarizability. By summarizing the theoretical model of SERS, SERS enhancement mechanisms were divided into two categories: the physical enhancement (electromagnetic enhancement) mechanism and the chemical enhancement (molecular enhancement) mechanism [25,26]. Electromagnetic enhancement plays a dominant role. The enhancement mechanism of chemical enhancement mainly includes: (1) The enhancement of Raman peak intensity by molecule bonding with metal; (2) Complex formation and the enhancement caused by charge transfer of metal molecules induced by laser [27]. The principle of electromagnetic enhancement is that when the roughened metal matrix surface receives light irradiation, the plasmon on the metal surface can be excited to the energy level, coupled with the electric field of light wave, and resonance, so that the electric field on the metal surface is enhanced, resulting in enhanced Raman scattering [28]. The main reason for electromagnetic enhancement is that the surface plasmon resonance induced by the collective oscillation of free electrons on the surface of noble metal nanostructures can greatly enhance the electromagnetic field on the surface of noble metal nanostructures [29]. Noble metal nanostructures have sharp structural characteristics, large curvature, and proximity to each other, which can obtain large electromagnetic enhancement at the tip or gap of nanostructures, especially at the gap due to electromagnetic field coupling. Such structures are called “hotspot” [30]. The accuracy of SERS has shown to be as good as that of liquid chromatography/tandem mass spectrometry, but the method has the added advantages of being non-destructive, simple, fast, and suitable for on-site detection [31].

SERS has unique advantages in practical application: (1) It has high sensitivity and is capable of single-molecule detection, which is often used for the detection of trace substances [32]; (2) The SERS signal could reflect the specific molecular fingerprint information of detected substances, with high specificity and selectivity [33]; (3) Compared with the traditional Raman spectrum, SERS is generated by the strong plasmon thermal effect of noble metal nanoparticles, which can improve the spectral resolution; (4) It has strong resistance to background fluorescence, water, and air, and compared with fluorescence detection, SERS is resistant to photobleaching and photodegradation, so it is suitable for long-term monitoring of substances [34]; (5) SERS-active nanostructures can be designed with different sizes, shapes, and substrates of coatings for different detection purposes; (6) SERS is suitable for the detection of gas, liquid, and solid samples.

### 1.5. Application of SERS in Food Safety Detection

Food safety is directly related to human health. It puts forward high requirements for the sensitivity, reliability, rapidity, and safety of detection methods. Raman spectroscopy has been widely used in food safety detection, including microscopic Raman spectroscopy, Raman imaging, and SERS. SERS has been continuously improved and improved in continuous experimental exploration, and it has been extensively applied as a powerful biochemical fingerprinting method [35]. Based on the advantages of SERS, its application in food field is very extensive, including quantitative analysis, qualitative analysis, and structural analysis; for example, (1) Basic ingredient detection; (2) Detection of toxic and harmful substances [36], including food additives, pesticide residues, heavy metals, microbial toxins, and food-borne pathogens [37]; (3) Microbial detection and identification [38].

In order to obtain the Raman spectra of different target molecules, many methods to enhance the Raman effect are explored: (1) The innovation of active substrates, including exploring the combination of different metal nanoparticles semiconductor and different active substrates sensor production, etc.; (2) The combination of substrates and samples; (3) Combined with sample pretreatment technology; (4) Combined with machine learning stoichiometry analysis data. Guo et al. [39] developed a fast and sensitive SERS method for detecting Pb^2+^ in food by modulating the aptamers reduced by gold nanoparticles. The method was successfully used for the detection of lead in tea with a detection limit of 0.1 μg L^−1^. Kashif et al. [37] through SERS combined with stoichiometric methods (including principal component analysis (PCA) and partial least squares discriminant analysis (PLS-DA)), proved to be an effective technology for identification and differentiation of food-processing bacteria. These innovations amplified the advantages of surface-enhanced Raman spectroscopy.

**Figure 4:**
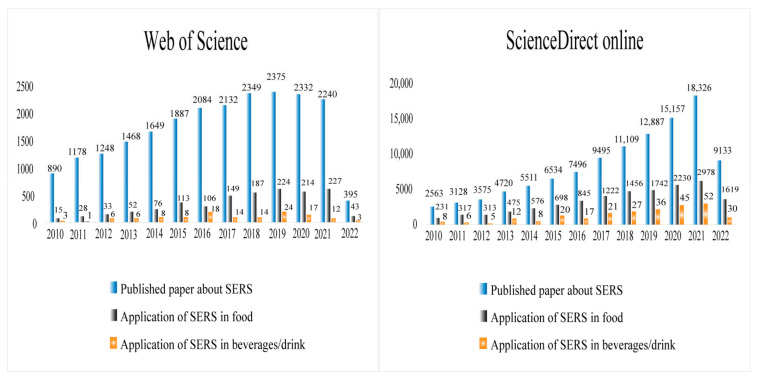
Statistics on the number of papers about SERS technology and its application published in Web of Science and ScienceDirect database in recent ten years (from January 2010 to April 2022).

The research statistics of SERS in food and beverage detection for the last decade have been summarized in Figure 4 (data were collected from January 2010 to April 2022). SERS has rapidly developed into an effective and widely used in food safety detection technology [2]. Alcoholic beverages refer to beverages with ethanol content above 0.5%VOL for people to drink, which are liquid food for people to drink directly. Therefore, as a special food, the development of SERS also provides new technical support for the safety detection of components of alcoholic beverages.

### 1.6. SERS in the Detection of Alcoholic Beverages

China is a big market with respect to the production and consumption of alcoholic beverages in the world. The production of alcoholic beverages in China has a long history and the technology has been passed down generations. In the long history of development, abundant varieties have been produced. Alcoholic beverages represent a broad category, with a wide range of alcohol content, including wine, beer, sparkling wine, liquor, fruit wine, and so on. The composition and safety testing of alcoholic beverages has become an extremely important stage in the import, export, and sales of the industry at home and abroad. As a liquid beverage, an alcoholic beverage may contain self-produced or artificially added toxic and harmful substances [40], such as food additives and food colorants [41], food adulterants, toxins [42], heavy metal [43] etc., which can be directly ingested and accumulated in the body, and ultimately affect human health [44]. However, there are the following difficulties in the component and safety detection of alcoholic beverages: the main component is water, the content of detection substances is low, and the components in wine are complex. Raman scattering provides another advantage over infrared radiation (IR), fluorescence, and other analytical techniques; namely, its ability to process samples with water as the main component. Existing studies have pointed out that SERS showed certain advantages in the detection of beverages, mineral water, alcoholic beverages, and other substances with high water content [45].

Many analytical methods include microbiological technology, microscope, cell plate, the colorimetric method, the titration method, and the enzymatic method to determine the main volatile components in alcoholic beverages. With the continuous development of science and technology, more advanced and sophisticated instruments are used to detect the active ingredients in alcoholic beverages. For instance, liquid chromatography-mass spectrometry (LC-MS) was used to detect anthocyanins, flavonoids, and polyphenols in wine [46], while the main volatile components were detected by GC-FID [47] and gas chromatography-mass spectrometry (GC-MS) [48]. Some low molecular dopants in alcoholic beverages were detected by headspace gas chromatography-mass spectrometry (HS-GC/MS) [49]. These methods are expensive, time-consuming, labor-intensive, and require complex sample pretreatment. Therefore, it is necessary to find suitable, rapid, and non-destructive methods to detect trace compounds in alcoholic beverages. For instance, real-time direct mass spectrometry (DART-MS), fluorescence spectroscopy, Fourier transform mid-infrared spectroscopy [50], Microextraction-Gas Chromatography-Mass Spectrometry (SPME-GC-MS) [47], two-dimensional gas chromatography combined with the time-of-flight mass spectrometry (GC × GC-TOF/MS) [51], and so on.

In the past few decades, vibrational spectroscopy has been widely used in the detection of alcoholic beverages [52]. SERS, as a research hotspot in recent years, can improve the signal of Raman technology by several orders of magnitude, combining high sensitivity and high specificity [2], and has been gradually applied in the field of food [53]. It also combines molecular fingerprint specificity with potential single molecule sensitivity [54]. Boyaci et al. [55] used Raman spectroscopy to directly quantify ethanol and methanol in distilled alcoholic beverages, and obtained Raman spectra of different ethanol–methanol mixtures. This method is sensitive, cheap, and fast, while simultaneously, the analysis time is less than 30 s. In addition, most of the measurement errors and interference are eliminated [55]. Since the main components of most alcoholic beverages are water (about 84%) and ethanol (about 12%), these two species, especially ethanol, dominate the normal Raman spectrum, and thus, greatly interfere with the detection of target components in alcoholic beverages.

SERS has the flexibility to design active substrates of different sizes, shapes, and coatings according to detection purposes and substances. This technique brings new hope to the field of non-destructive testing and has great potential in the analysis of alcoholic beverages. Based on the above advantages and developments, this technology has been proved to be feasible for non-destructive testing of beverages [56] and alcoholic beverages [45]. Although FT-Raman spectroscopy is suitable for fluorescent species, its main disadvantage is that the signal of the diluted compounds contained in the sample is weak [57]. SERS technology is an effective fluorescence quenching method [57].

**Figure 5:**
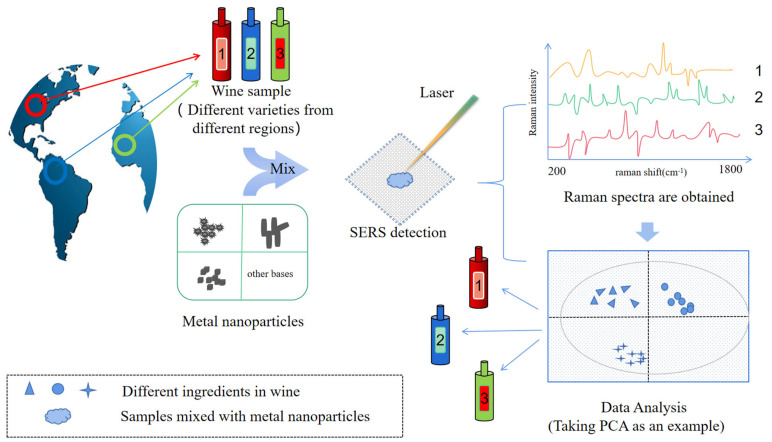
The SERS technique was used to identify and characterize different wines from different regions. In this figure, three kinds of wines from different regions and different varieties were selected, and Raman detection was carried out by combining metal nanoparticles. Then, the Raman spectra of different wines were analyzed by principal component analysis software to realize the recognition and characterization of different wines.

**Figure 6:**
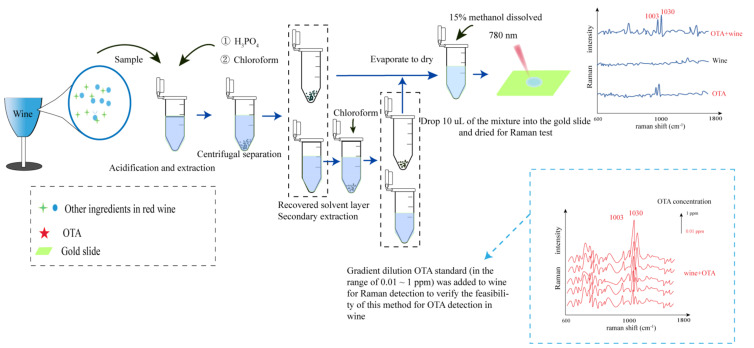
Illustrative diagram of SERS conjugation liquid–liquid extraction technology for the detection of trace harmful substance Ochratoxin A in wine.

Many studies have shown that SERS has been successfully applied in the detection and identification of alcoholic beverages; for example: (1) To distinguish and identify alcoholic beverages through composition testing, where experimental results show that surface-enhanced Raman spectroscopy is more successful in the identification of origin and variety of white wine [58]. Take Figure 5 for example; combined with machine analysis, different red wines were distinguished according to their composition [59]. (2) Detection of nutritional components and active ingredients in alcoholic beverages; due to its chemical specificity, SERS was highly sensitive to variations in molecular composition, and provided valuable information for components of anthocyanins, flavonoids [60], polyphenols, resveratrol, etc. Magdas et al. [61] studied the stability of cheap wine and the interface between AgNPs by wet chemical reduction method for the first time. The experimental results show that the technique can selectively enhance the secondary compounds (anthocyanins and phenolic acids, etc.) [61]. (3) Detection of toxic and harmful substances. There are many studies on the detection of self-produced toxic substances from additive adulterants in food. It has been proved that SERS has many advantages in the detection of these substances, such as sensitivity, speed, trace amount, quenched-fluorescence, and low water interference [2]. SERS is more cost-effective and simpler. Bettini et al. [62] designed a SERS detection method for silver nanoparticles (AgNPs)–cellulose hybrid substrate through experiment. The results showed that histamine could be detected in 10–12 M of aqueous solution, and the method was directly tested on commercial white wine, and the sensitivity of the device was confirmed. Taking Ochratoxin A in red wine as an example, mycotoxins in red wine can be quickly detected with a detection limit of 0.01 ppm through liquid-liquid extraction combined with nanoparticles (Figure 6) [63]. (4) Microbiological testing. There are many related microorganisms in alcoholic beverages, including beneficial microorganisms that assist brewing and harmful microorganisms produced in the production process [64]. Lemma, T. et al. used surface-enhanced Raman spectroscopy (SERS) and helium ion microscope (HIM) to detect three kinds of yeasts, and obtained the SERS spectra of the three kinds of yeasts in beer culture medium. The results showed that highly enhanced and repeatable Raman signals were generated and it was possible to distinguish the three species based on their unique characteristics (spectral fingerprints) [65]. Therefore, the potential of SERS technology in microbial recognition, detection, and identification is also worth paying attention to [33].

In recent years, SERS has been applied to the analysis of alcoholic beverages. Arslan et al. summarized non-destructive testing technologies for alcoholic beverages. Raman spectroscopy shows great potential due to its ability for on-site detection, quantification, identification, and classification [45]. Arroyo-cerezo et al. reviewed the practical application of spatially shifted Raman spectroscopy for food and beverage analysis, emphasizing the importance of Raman spectroscopy in multivariate analysis [66]. Zeren et al. reviewed the detection limits of methanol and ethanol content in alcoholic beverages by GC/MS and Raman spectroscopy, highlighting the advantages of Raman spectroscopy as an inexpensive, rapid, and non-destructive technique [67]. According to statistics, there have been relevant reviews on the application of SERS in food and other fields [2]. In a recent study, Bunaciu et al. reviewed recent applications of vibrational spectroscopy in the analysis of alcoholic beverages [68]. However: (1) The innovation direction of SERS technology in the field of food, including the detection of alcoholic beverages, has not been summarized; (2) The application of SERS technology in alcoholic drinks has not been summarized and discussed. This paper briefly summarizes the innovative development direction, advantages, and development potential of SERS technology in the field of food and alcoholic beverages, summarized the components in alcoholic drinks and the significance of component detection, and mainly summarized the practical application of SERS technology in the detection of compounds in alcoholic drinks. This paper will provide a reference for the innovation and development potential of SERS technology in the future, and will also stimulate the innovation of SERS technology in the future alcohol detection, and constantly explore rapid on-site detection methods.

## 2. Latest Development of SERS Technology

### 2.1. The Development of Substrates in Raman Technology

In recent decades, researchers have expanded on the theory of SERS technology, identified the limitations and defects of this technology, and developed substrates conducive to SERS technology. At present, materials used as SERS-active substrates mainly include noble metal nanomaterials (Au, Ag, etc.) [28] and non-metallic nanomaterials (semiconductor graphene, SiO_2_ nanomaterials, Graphene-titanium dioxide composite nanomaterials, Ferric oxide @ graphene oxide @ titanium dioxide (Fe_3_O_4_@GO@TiO_2_) nanocomposites [69], etc.) [70]. Researchers found that SERS enhancement could be optimized by reasonably designing and controlling the size, shape, and material of nanoparticles [71]. There are many ways to prepare SERS-active substrates [72]. In addition to various nanosubstrates mainly prepared by noble metal nanomaterials, there are also innovative studies on substrates with positive orientation, such as the addition of gold, silver, and other nanoparticle sol drops to silicon or glass sheets to form a metal film with SERS activity [73]. In addition, SERS substrates could be directly modified on the surface of silicon or glass. At present, silicon and glass wafers are used as SERS substrates, as well as simpler and more accessible substances, such as metal wafers, paper, and capillary tubes. Over the past few decades, there has been continuous exploration and innovation of the preparation of nanoparticles of various types and shapes, such as nanorods [74], nanocubes, nanostars, nanosheets, nanotriangles, and nanowires [75]. In addition, some substrates have been obtained by special assembly [76].

#### 2.1.1. Colloid Substrates

Metal nanostructures have taken on many different forms to adapt to the detection of different substances, and these structures must be stable and cost-effective to ensure feasibility of the SERS platform. Among them, gold and silver are the most commonly used SERS substrates compared with other precious metals (lithium, sodium, nickel, copper, etc.). Researchers synthesized nanoparticles or colloids through different methods to increase the generated Raman signal. For Ag and Au colloidal solutions, Zhang et al. examined the role of nanoparticle surface charge in SERS and found colloidal nanoparticles. The combination of silver colloids increased the Raman signal for the detection of melamine by at least 10^5^ times. The method was simple, fast (only about 3 min), economical, and efficient, as well as sensitive to the detection of melamine in liquid milk samples [31]. For the detection of organic dyes in water, Xue et al. [77] employed silver nitrate water thermal reduction for the preparation of nanometer silver sol. The nanometer silver hydrosol had a large particle size, wide distribution, good dispersibility, and spherical shape. This material led to enhanced Raman signals of three organic dyes [77]. Xiao et al. used light trace Raman 202 (OTR 202) as a SERS-active colloid in their study.

#### 2.1.2. Solid Substrate

To adapt the nanoparticles to the needs of different environments and achieve rapid detection of trace analytes in the field, researchers are looking for many ways to protect and recombine the nanoparticles to form a variety of substrates and protectors. The solid substrate can form a flat surface so that the target molecules are arranged in an orderly manner on the substrate, which generated a uniformly distributed Raman signal, as well as improves reproducibility of the SERS spectrum. Yoon et al. prepared the substrate by fixing Ag colloidal particles on a glass plate coated with poly (4-vinylpyridine) (P4VP), adsorbing and fixing molecules on the fixed colloidal particles. Then, weak adsorption molecules, such as aniline, were used to replace the target molecules for adsorption, and a simple SERS platform with high Raman signal enhancement was obtained [78]. The preparation and analysis of a paper-based SERS sensor were demonstrated by inkjet printing or dropping colloidal AuNPs onto paper. The solid substrate meets the criteria of stability and economic efficiency, and it has broad applications for a variety of samples [79]. Cheng et al. used a highly uniform AgNR array substrate to perform SERS detection of melamine in real feed samples. The active AgNR array substrate was prepared in a customized electron beam evaporation system using angle deposition (OAD). Arrayed nanorods with lengths of ~900 nm and diameters of ~100 nm were obtained, and the substrate exhibited high sensitivity [80]. Camerlingo et al. [81] designed a teller mi-based plate, which consisted of a glass slide coated with AuNPs 30 nm in diameter. The substrate is low-cost and relatively easy to prepare, requiring no significant investment of capital or technology. A key aspect of SERS application in industrial applications is to obtain simple and low-cost SERS substrates, which have minimal chemical consumption and time requirements and can even be routinely used in production lines [81]. Therefore, highly ordered solid SERS substrates usually have better signal reproducibility, compared with colloidal substrates. These solid substrates make the active substrate more controllable and stable. Future directions of SERS technologies include the detection of samples with a weak Raman signal and the design of specific solid substrates with high selectivity and low cost.

#### 2.1.3. The Flexible Base

Traditional solid SERS substrates, based on silicon, glass, and porous alumina, have some limitations, such as isomorphism, rigidity, and brittleness. Thus, the development of a flexible substrate has been explored. Flexible SERS-active substrate refers to the construction of plasmon nanostructures with SERS activity on flexible solid scaffolds; compared with solid substrates, this nanostructure has the advantages of convenient use and low cost [82]. Flexible substrates can overcome the disadvantage of rigid substrates not being able to adhere to rough, irregular sample surfaces. The flexible substrate has good environmental adaptability; therefore, it provides a new method for non-invasive or minimally invasive sample detection. Moreover, it is of great significance for fragile and high-value samples [83]. In 2017, He et al. [84] developed a paper-based SERS substrate, on which gold nanostars were immobilized. The device had the advantages of portability, low cost, and high sensitivity, and it could be fabricated as a commercial SERS substrate. Hang et al. [85] first proposed a gecko-inspired nanotentacular SERS (G-SERS) platform to detect three pesticide residues. The G-SERS substrate provided excellent SERS activity (enhancement factor = 1.2 × 10^7^), excellent reproducibility (RSD = 5.8%), and numerous flexible nanoscale ‘tentacles’. At present, detection sensitivity is the main challenge in developing a paper-based SERS. The fibrillary structure of the paper material, the ability to modify and improve the paper-metal interface bonding, and the surface conditions of the metal nanostructure all contribute to the SERS performance of the paper device [85]. Wang et al. used the capillary effect of filter paper and the rapid detection of SERS technology to prepare a SERS multifunctional sensor card for the detection of food contaminants plasticizer butyl phthalate (BBP) in liquid, and thiabendazole (TBZ). The technique utilized a liquid–liquid interface and self-assembly method to synthesize gold nanotriangles and prepare dense nanomembranes with large surface area. The nanomembranes were transferred to the surface of filter paper to create SERS-based membranes [86].

#### 2.1.4. Mixed Nanomaterial Substrate

Based on SERS substrates with high activity, compound fingerprint recognition can be realized. Mixed nanomaterial substrates have advantages in the detection of some substances. Some studies have shown that by mixing different materials [87], the Raman signal of target molecules on the substrate surface can be enhanced to achieve sensitive detection of target substances [88]. In order to achieve rapid SERS analysis of target substances in complex matrices, a high-activity enhanced substrate is needed. Cheng et al. proposed a fast, reliable, and quantitative method for the determination of acrylamide content in fried food based on a silver nanorod/gold nanoparticle composite material (AgNR@AuNP) SERS. Based on the double ‘hotspot’ enhancement effect of AgNR nanorods and AuNPs, the substrate had a high SERS enhanced activity for acrylamide, which greatly reduced the detection time of acrylamide [89]. As a new plasmon material, TiN had strong SERS performance, good chemical stability, and biocompatibility, but its SERS performance was not as good as gold and silver. Therefore, Wu et al. prepared Au/TiN composite films by depositing Au nanoparticles on the TiN film surface by ammonia nitrate reduction and electrochemical deposition. The number and size of metallic AuNPs on the surface of the TiN film increased gradually by electrochemical deposition, which verified that the Au/TiN composite film has a particularly strong enhancement effect [90]. In addition, the combination of non-metal and metal materials is also a popular SERS research topic. A composite of graphene and metal nanostructures produced strong electromagnetic enhancement, and the graphene layer could be placed on a SERS planar substrate. The graphene layer is attached to the metal surface so that the molecules can be orderly arranged on the substrate surface. Moreover, it separates the metal nanoparticles and molecules, which can exclude the signal interference that may be caused by the interaction between metal nanoparticles and molecules [91]. The combination of the Raman enhancement effect of nanomaterials and the enrichment ability of the graphene layer improved the sensitivity of the substrate [92]. In 1983, Yamada and Yamamoto [93] prepared SERS-active material by depositing some metal on the surface of NiO and TiO_2_. Research on composite substrates containing non-metallic nanostructured materials has been increasing, such as on the Titanium dioxide nanostructure [94] and silicon nanowire structure [95]. SERS substrates of this kind of composite semiconductor materials have made significant progress in some applications of SERS spectrum. They have additional important functions, which cannot be obtained by standard SERS substrates produced by plasmon metals only.

#### 2.1.5. Semiconductor Substrates

Despite the analytical advantages of metals and nanometallic substrates, the production of these materials is costly, and there remain deficiencies in the detection of some substances. Therefore, some new semiconductor materials with unique chemical structures and physical and chemical properties have been applied in SERS. For example, graphene, titanium dioxide, ZnO, Si, and other two-dimensional inorganic materials [96] (transition metal disulfide (TMDC), hexagonal boron nitride (H-BN), black phosphorus (BP), and MXenes) have been explored. Among them, semiconductors or insulators and their nanostructured surfaces are often used as SERS substrates. The production cost of these substrates is mostly lower than that of standard SERS substrates made of pure gold or silver. In 1982, Loo recorded SERS spectra with polycrystalline TiO_2_ as SERS-active material and observed the SERS effect on the semiconductor surface for the first time [97]. In 2011, Yang et al. prepared molybdenum oxide and graphene oxide (MoO_2_/GO) nanocomposites through hydrothermal assisted synthesis, and the experimental results showed that the nanocomposites exhibit high stability, reproducibility stability, and acid and alkali resistance [98]. Semiconductor nanomaterials have excellent surface structural stability, high molecular selectivity, and high biocompatibility. They are not easily oxidized or aggregated, and there is great potential for development. Graphene [99], with sp^2^ hybrid-connected carbon atoms tightly packed into a single two-dimensional honeycomb lattice structure, is considered the gold standard. It has a large surface area, smooth surface, high stability, and unique electronic/optical characteristics. Thus, it has great development potential as a SERS substrate [91]. In 2010, Liu et al. first discovered the Raman scattering phenomenon in graphene materials [100]. With the continuous exploration of SERS technology, substrates based on graphene have been continuously applied, including combinations with other materials to generate a low-cost, highly sensitive, and stable structure [101]. Semiconductor substrates can selectively detect specific molecules even in complex mixtures. Moreover, if the choice of substrates is extended to organic semiconductors, the ability to refine the resonance locations of charge transfer can be improved. In a recent study, Yilmaz et al. [102] used π conjugated organic substrates composed of α, ω diperfluorhexyltetrathiophene (DFH-4T) to form a nanostructured film that strongly enhanced the Raman signal of methylene blue. Lombardi [103] extended the unified theory of surface-to-enhanced Raman spectroscopy to semiconductor substrates composed of organic monomer aggregate, and the results showed that compared with inorganic semiconductors, the J-aggregate exciton band is quite narrow, usually equivalent to the laser width, improving the applicability of this technology.

#### 2.1.6. Substrate Preparation by the Top-Down Technique

The top-down approach is used to prepare various metal nanomaterials through some physical methods or micro-machining methods. Common methods include electron beam photolithography [104], argon ion sputtering [105], and focused ion beam micromachining. One of the most widely used techniques is electron beam lithography. These methods can produce large-scale metal nanomaterials with controllable shape, uniformity, and good reproducibility. However, they also have the disadvantages of complex operation and high cost. SERS ‘hotspot’ of base metal nanomaterials obtained by top-down technology were relatively uniform, which ensured the reproducibility and stability of the spectra detected by SERS technology. Li et al. reported a lithography and chemical displacement method for the preparation of AuNPs with a square array pore structure. AuNPs were immobilized in the square lattice of the SERS substrate, which was large in size and showed high enhancement, high repeatability, and recyclability. The results show that the AuNPs produced by this method are denser and more orderly; thus, longer and stronger SERS signals can be obtained [106].

#### 2.1.7. The Bottom-Up Technique Was Used to Prepare the Substrate

The bottom-up method mainly uses Au, Ag, Cu, and other noble metal nanounits to form various nanomaterials through self-assembly, such as nanoparticle self-assembly and the gel method. By changing the reaction conditions, precious metal nanomaterials of various shapes, such as polyhedrons, spheres, and cubes, can be formed. The bottom-up method is easy to operate and realize. In 2009, Huang et al. controlled the density of silver and gold nanoparticles by changing the volume ratio of the gold and silver colloids, and the authors self-assembled Ag and Au nanoparticles on silicon substrate simultaneously. Through experimental verification, the authors found the spectra of the assembled nanoparticles were similar to those of pure Ag and pure Au. Thus, the coupling effect between Ag and Au nanoparticles was proved [107]. In 2013, Ma et al. realized trace detection of antibiotics in water by using a silver colloidal film, prepared by a self-assembly method, as a SERS-active substrate. The substrate exhibited high sensitivity and application value [108].

### 2.2. Direct Detection Technology of SERS

**Figure 7:**
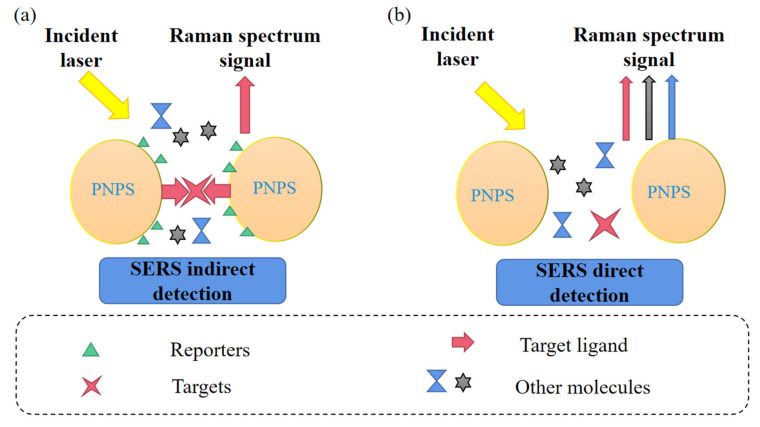
SERS direct and indirect detection technology. (**a**) The active substance in the sample was in direct contact with the metal nanoparticles, and the Raman signal came from molecules with high enhanced activity. (**b**) The nanoparticle binds to the target ligand with specific recognition function, and the target substance in the sample binds to the ligand to form a strong hotspot and recognize the target molecule.

SERS direct detection technology, also known as unlabeled detection technology, uses the target molecule and metal surface bonding to measure the object near the surface of the substrate and to enhance the target molecule Raman signal (Figure 7). Using the sensitive and rich fingerprint spectrum properties of SERS, the characteristic single spectral band (molecular fingerprint) of the fingerprint area of the target analyte can be analyzed. This method has the advantages of simple operation and simple data analysis [109]. Continuous development of highly sensitive, biocompatible, or reproducible SERS-active substrates has become the focus of unlabeled SERS research. Wang et al. proposed a simple, fast, and unlabeled SERS mapping method that utilized the inherent and unique SERS signals of single bacterial cells to conduct unlabeled detection and target differentiation. The signal was two orders of magnitude lower than the detection limit obtained by the traditional SERS method under the same experimental conditions [110].

### 2.3. Indirect Detection Techniques for Surface Enhancement

SERS indirect detection technology, namely, marker detection technology [34], is a detection method mainly for ions and small molecules with small scattering cross-sectional areas. The Raman activity of organic molecules is combined with that of the target to enhance the Raman signal and improve the sensitivity. The target analyte is not in direct contact with the metal surface but rather with the organic molecules, such as Raman dyes (Figure 7). To optimize indirect detection methods, multifunctional SERS markers must be designed and developed with high sensitivity, specificity, and selectivity. SERS signals are observed only when the target molecule combines with the organic ligand or the SERS markers respond to environmental changes, such as pH, ion concentration, and temperature, to indicate the presence of the target biomolecule. Recognition molecules, such as specific antibodies and adaptors, have been modified on SERS substrates, which had two functions: first, appropriate biocompatibility was given to SERS probes; second, the SERS probe was endowed with the targeting function and specific recognition target. The presence of the target molecule or the physical or chemical properties of the sample can be indicated by observing the specific Raman spectrum or spectral variation of the marker [34].

Yazgan et al. used spherical magnetic-core gold-shell nanoparticles and rod-like gold nanoparticles (nanorods) labeled with Raman active compounds to form complexes with melamine molecules. These structures achieved sensitive and rapid detection of melamine in food [111]. The combination of SERS with immunochromatography (ICA) is commonly used for indirect detection. Li et al. [112] used AuNPs as a SERS substrate, and fluorescence-labeled AuNPs were used to generate the Raman signal. Through the immunospecific binding of antigen and antibody, the specific recognition and detection of SERS to the target substance was promoted. Hughes et al. proposed a novel nanosensor for detecting ultra-trace bioactive molecules in complex matrices. The nanometer sensor by antibodies with thin silica shell and surface of AuNPs attached objects, allowed by SERS, used fixed and direct detection of biologically active molecules, and the adhesion on the surface of the nanoparticles, antibodies with high affinity to selectively target molecules, sensor, and the target analyte interaction. The Raman spectra generated from the surface effects of nanoparticles were significantly enhanced [113].

Compared with SERS direct detection, SERS indirect detection has the advantages of high specificity, accuracy, and reduced interference. It is often used in biology, cytology, and life science-related technical fields.

### 2.4. SERS Combined with Machine Learning and Multivariate Analyses

Statistical analysis discovery of SERS was used for qualitative and quantitative analysis of target substance. Therefore, the combination of machine learning and multivariate statistical analysis can play an effective auxiliary role. For example, researchers used the density functional theory (DFT) to calculate the Raman spectrum of melamine, and the results were in good agreement with the spectrum calculated by DFT [80]. Sun et al. used SERS technology combined with linear regression algorithm for the rapid quantitative analysis of pesticide residues in honey. The relationship between Raman displacement intensity and dimethoate pesticide residue concentration was established through the linear regression algorithm, and the concentrations of 10 samples were predicted in the evaluation model. By comparison, the optimal model peak shift was 867 cm^−^^1^. The authors reported a high prediction correlation coefficient of 0.984 and a low prediction root mean square error of 0.663 ppm, indicating the method could quickly detect pesticide residues [114]. Machine learning is used to reduce the workload and the possibility of artificial misjudgment. Principal component analysis (PCA) and support vector machine (SVM) were used to verify the advantages and feasibility of this method in the identification and quantification of flibanserin in liquor, beer, and wine. This method fully demonstrated the huge application potential of SERS technology combined with machine learning in the rapid on-site detection of psychedelic drugs [115].

### 2.5. SERS Combined with Sample Pretreatment

When SERS was used to detect substances with low concentration and complex composition, some experiments showed poor detection sensitivity, long detection time, weak Raman signal, interference, and other shortcomings. Therefore, several sample pretreatment methods have been incorporated, including microchip capillary electrophoresis, dispersed solid-phase extraction, liquid–liquid extraction, and in situ microextraction. Cheng et al. developed a fast, reliable, and quantitative method for the determination of acrylamide content in fried foods based on SERS via GO/gold nanoparticle composites and dispersed solid-phase extraction. Using this method, the measurement time of each sample was reduced, which proved the use of dispersed solid-phase extraction improved SERS detection [116]. Polycyclic aromatic hydrocarbons (PAHs) can migrate from contaminated food contact materials to food. In a recent study, SERS, and surface microextraction were combined for in situ screening of PAHs on food contact materials. The method rapidly screened PAHs on contaminated food contact materials without complex sample pretreatment [117]. Microchip capillary electrophoresis and SERS were employed for the identification of vibration fingerprint spectrum, with high specificity and efficiency. The method was successfully applied to the determination of riboflavin in barbecue sauce [118].

### 2.6. SERS in Combination with Other Techniques

Although SERS has greatly improved the detection sensitivity and speed through the innovative combination of various nanomaterials, samples in real life usually contain various complex components, which also create challenges in detection. The combination of other technologies can not only overcome these limitations, but also enhance the detection and characterization ability of SERS [53]. For example, chemical separation, biological capture, colorimetry, microfluidic devices, and headspace film microextraction (TFME) [119] have been combined with SERS to enhance detection capabilities. In addition, the combination of SERS and other chemical analysis techniques, such as mass spectrometry, nuclear magnetic resonance, infrared spectroscopy, and X-ray photoelectron spectroscopy, have led to more accurate characterization [120].

Wu et al. developed a specific and sensitive patulin (PAT) sensor based on molecularly imprinted polymer (MIP) and SERS technology. AuNPs have good SERS characteristics, and MIPs exhibit excellent selectivity. Therefore, the MIP-SERS sensor had excellent performance in PAT detection. Under the optimized conditions, the sensor showed good linearity (R^2^ = 0.9877) for PAT concentrations from 7.00 × 10^−12^ to 5.00 × 10^−8^ M. Even in the case of multiple interferences, the MIP-SERS substrate showed good selectivity to PAT [121].

Gao et al. [121] proposed the first three-mode method for on-site identification and detection of trinitrotoluene (TNT) using colorimetric, fluorescence, and surface-enhanced Raman scattering (SERS) responses of AuNPs @quantum dots (GNPS-QDs) core-satellite components. This method verified the reliability of quantitative determination of TNT in soil, clothing, fruit, liquor, and other environmental samples [122]. The surface swab capture method combined with SERS was used for the recovery and quantitative detection of thiabendazole on apple surfaces. The swab-SERS method was simple, sensitive, fast, and quantitative, and it was sufficient for QA/QC in commercial settings [123].

The analysis of real samples by SERS sensors can be challenged by the complexity of the sample matrix. Thin-layer chromatography (TLC) is a promising technique for the detection of substances in complex samples because of its simple operation, low cost, and fast separation time. Combining TLC with SERS can lead to high throughput and sensitive analysis [124].

## 3. Ingredients of Alcoholic Beverages

### 3.1. Basic Ingredients of Alcoholic Beverages

Alcoholic beverages include fermented wine, distilled wine, and prepared wine [125]. According to the summary, in addition to the main components of water and ethanol, there are other components, including basic components (aroma and taste substances), nutritional active components, toxic and harmful components, microorganisms, etc. This part introduces the composition of alcoholic beverage and its effect on alcoholic beverage and human body. Table 1 summarizes the proportion and effects of relevant ingredients in alcoholic beverages.

Alcoholic beverages contain rich aroma components, including esters, alcohols, acids, aldehydes, carbonyl compounds, nitrogenous compounds, sulfur-containing compounds, furans, ethers, phenols, amino acids, and other substances [126]. By 2017, a total of 1874 compounds had been identified in alcoholic beverages, and by August 2019, more than 2000 compounds had been reported [127]. These compounds directly or indirectly affected flavor quality characteristics. The quality characteristics of commercial alcoholic beverages have been evaluated by pH, total acidity, acetaldehyde, SO_2_, and flavonoid content. The pH value of diluted soju wine was the highest, and the total acidity and flavonoid content of fruit wine was higher. SO_2_ has been detected only in red and fruit wines [128]. The main organic and inorganic components of wine include sugars and related simple carbohydrates, alcohol, organic acids (fixed and volatile), phenolic compounds (anthocyanins, non-flavonoids, and tannins), carbonyl compounds (aldehydes and ketones), ester, lactones, and other heterocycles, terpene, nitrogen compounds, mercaptan and other organic sulfur components, hydrocarbons and their derivatives, macromolecules (carbohydrates, lipids, proteins, nucleic acids), vitamin, dissolved gases (carbon dioxide, sulfur dioxide, oxygen), and minerals [129]. Fruit wines are known for their health-promoting properties and are prized for their taste and active ingredients. Fruit wine are rich in polyphenols with biological properties, such as anti-oxidation and anti-cancer activities [130]. Cakar et al. investigated the effect of fruit wine on the prevention of diabetes and other chronic diseases, analyzing the fruit wine made from blueberry, bitter berry, blackberry, raspberry, and sour cherry. Compared with acarbose used as a positive control (IC50 = 73.78 μg/mL), all fruit wine samples showed higher α-glucosidase inhibitory activity. It is concluded that phenolic substances in fruit wine enhance the application value of fruit wine [131].

### 3.2. Beneficial Alcohol Ingredients

Alcoholic drinks are associated with the development of several chronic human diseases, such as cancer, cardiovascular disease, diabetes, and obesity [132]. In recent years, more attention has been paid to the influence of alcohol on human health. As a result, many researchers and wineries aimed to make wines with more health benefits and to reduce the side effects of alcohol on the human body. Many of the active ingredients in the wine are extracted to meet the needs of special groups and brewed into healthy wines with certain effects. For example, 722 metabolites were identified in Tartary buckwheat wine, of which 84 are key active components of traditional Chinese medicine. In total, 78 are active components of six kinds of drugs for disease resistance. They are rich in flavonoids and trace elements that can be used to prevent cancer and cardiovascular diseases [133]. Zhuyeqing liquor (ZYQL) [134] and some Chinese medicinal materials with rich active ingredients have been made into a series of low-proof health wines. Therefore, the developed wine products have beneficial ingredients to improve health. Koguchi et al. found that the beverage made from purple rice was rich in anthocyanins. Purple rice was used to make alcoholic drinks, which contained 11.0% to 11.5% ethanol (*v*/*v*) and high amounts of phenolic compounds and antioxidant activity [135]. Wine contains many unique beneficial ingredients (anthocyanin, resveratrol, tannin, amino acids, vitamins, trace elements), as well as antioxidants (phenolic compounds, tannin, flavonoid compounds). Studies have shown that wine can prevent cerebral thrombosis, reduce cholesterol, prevent atherosclerosis, and regulate intestinal function. It also has anti-aging, anti-cancer, and cardiovascular protective effects [136]. Resveratrol, a natural ingredient found in wine, has been identified as the main ingredient in these health promotions. Resveratrol has many valuable properties, such as anti-aging, cardioprotective, and anti-cancer activities [137].

### 3.3. Toxic and Harmful Ingredients

Numerous harmful ingredients have been found in the brewing, transportation, and storage of alcoholic beverages, such as adulterated substances, food additives, harmful substances integrated into packaging, illegal additives, by-products of long-term storage, and microbial contaminants. Pflaum et al. summarized and discussed 18 carcinogenic compounds (acetaldehyde, acrylamide, aflatoxin, arsenic, benzene, cadmium, ethanol, ethyl carbamate, formaldehyde, furan, glyphonate, lead, 3-MCPD, 4-methylimidazole, N-nitroso dimethylamine, pulegone, ochratoxinA, safrole) in alcoholic beverages. Among them, ethanol can solubilize various carcinogens or precarcinogens in alcoholic beverages. Moreover, in 2010, ethanol and its metabolite, acetaldehyde, were listed as human carcinogens [40]. Acetaldehyde, a chemical found naturally in food, imparts a subtle fruity flavor. Studies of several alcoholic beverages on the market have shown that the amount of acetaldehyde is positively correlated with the alcohol content. High levels of acetaldehyde can increase or alter the taste of food and drinks; however, acetaldehyde exposure may lead to cancer [138]. Cumulative risk assessments show the risks of ethanol and metabolized acetaldehyde greatly outweigh the risks of acetaldehyde directly contained in beverages [139]. With the increase in the types of alcoholic beverages on the market, many consumers cannot accurately identify the types and characteristics of alcoholic products. In addition, the alcoholic beverage industry is often affected by the addition of illegal adulterants and food additives in products. In 2010, Magnusdottir et al. found that the contents of methanol or ethylene glycol in alcoholic beverages were of particular concern. Methanol has been illegally added to wine to enhance taste, and ethylene glycol was added to sweeten the wine [140]. Studies have found that some microorganisms can also produce harmful substances that affect the quality and safety of alcoholic beverages. Nuruk is a traditional Korean fermentation product that is mainly produced by solid fermentation of wheat, rice, and other grains, and it is used as a starter for starchy alcoholic drinks. It contains a variety of microorganisms, and most of these microorganisms are useful to produce alcoholic beverages. However, Nuruk can be contaminated with *Aspergillus*, which produce aflatoxins (AF) that is harmful to the human body [42].

### 3.4. Microorganisms

The role of microorganisms in alcoholic beverages cannot be underestimated, as microbial fermentation technology is required for the development of alcoholic beverages. Yeast and lactic acid bacteria are the main microorganisms in fermented beverages [141]. Fermentation typically involves *Firmicutes* bacteria, filamentous fungi, enzymes, and alcohol-producing yeasts [142]. There are many microorganisms in alcoholic beverages, among which *Saccharomyces cerevisiae* is the most used microorganism in alcoholic beverage fermentation, especially in the process of spontaneous fermentation without starter. These microorganisms affect the sensory quality of alcoholic beverages and also affect product safety [143]. Some microorganisms produce toxic byproducts, including aflatoxins [42].

## 4. Application of SERS in the Detection of Alcoholic Beverages

### 4.1. Characterization and Classification of Alcoholic Beverages

Alcoholic beverages differ from one another and within each product category, due to the origin, storage time, the selection of raw materials, the manufacturer, and brand. High-resolution separation techniques, such as liquid chromatography, gas chromatography, mass spectrometry, or nuclear magnetic resonance spectroscopy, are most often used for the classification, certification, and authenticity of alcoholic beverages [144]. Studies have proved that the SERS spectral spectrum obtained was derived from the chemical components (such as metabolites) adsorbated on metal nanoparticles. There were significant differences between the spectra of different alcoholic beverages, corresponding to the differences of relevant components, so it could be used for characterization and even identification of alcoholic beverages [145]. The ability of SERS to determine molecular fingerprint spectra has proved useful in the identification of wine products [58]. In 2018, Magdas et al. reported the characterization of Romanian and French white wines using SERS technology. They examined 30 wines from three regions of Romania (Transylvania, Banat, Moldova) from 2011 to 2015. The identification rate of Feteasca Regala and Sauvignon varieties was above 90%, the geographical origin identification rate of Romanian varieties was 83.3%, and the separation rate between French and Romanian samples was 100% [61]. Zanuttin et al. [145] performed a further analysis on 180 different samples, based on the above research and combined with the SERS spectra and multivariate data analysis of non-resonant and unlabeled SERS stimulated by near-infrared light. It was shown that the main spectral differences were caused by adenine, carboxylic acid, and glutathione, which made it possible to distinguish wines from different provenances. Different wines could be distinguished according to their composition with an efficiency of 87% to 93%. Eight wines had a sensitivity of 75% and a specificity of 100%. The sensitivity of this method is low in distinguishing production years [145]. The research shows the advantages of SERS technology to distinguish the manufacturer, category, origin, and even year of wine products. To characterize the flavor differences in wine, Leong et al. [146] designed a machine learning-based SERS taster, in which the receptors used a variety of non-covalent interactions to capture the chemical functions of flavor molecules. By strategically combining the SERS spectra of all receptors, the vibration information from multiple receptors was used to enhance the detection of flavor molecules from five wines. Thus, multi-flavor quantification was achieved with high accuracy [146]. Table 2 summarizes the literature on alcoholic beverage characterization and parameter adjustment.

### 4.2. Detection of Beneficial Ingredients

Many alcoholic beverages of non-destructive testing technology development, such as UV, IR, fluorescence [149], nuclear magnetic resonance (NMR) spectroscopy, electronic nose and electronic tongue, etc. SERS solves the problem of these technologies cannot overcome, including the cost of restrictions, the influence of alcohol and water, single detection components, complicated sample pretreatment, not suitable for site test, etc. [45]. SERS can realize sensitive detection of different components through different ways, which has a great development space.

The chemical composition of wine is closely related to grape variety and wine quality. Therefore, the appearance of different Raman spectral peaks indicates differences between wine compositions. In 2018, Pinzaru et al. used silver nanoparticles (AgNPs) obtained by the classical wet method as the SERS substrate to evaluate the potential of SERS in the recognition of specific molecular components in wine. In the experiment, the authors identified the Raman spectral peaks of wine (red wine or white wine) at different times and contents. Combined with the analysis of flavonoid and phenolic substances in wine, the difference between white and red wine was obtained, which proved that this method can realize the rapid analysis and identification of wine [150]. To analyze the chemical components in red wine, a solvent extraction method combined with SERS technology was developed based on the original Raman spectroscopy to provide more characteristic information of wine. The characteristic spectral peaks of wine extracts matched those of condensed tannin, resveratrol [147], anthocyanin, gallic acid, and catechin. The use of SERS, in combination with solvent extraction, is an innovative method to analyze wine composition and overcome the challenges of analysis in red wine. Based on this method, three red wines were successfully distinguished, and the possible relationship between the peak intensity of spectrum and wine grade was proved [148]. In addition, there are some aromatic and flavorful volatile compounds in alcoholic beverages, such as formaldehyde (FA), acetaldehyde (AA), ethyl acetate, butyrate, and ethyl butyrate vary between species and content. Either too much or too little of these aromatic substances will affect the quality of alcoholic beverages. Therefore, the detection of these substances is necessary. Duan et al. [151] prepared AgNPs (Ag(NH_3_)^2 +^ complexes via aldehyde-induced silver reduction reaction with the addition of FA or AA as active substrates in a special way. This AgNPs had strong SERS activity, and the average particle gap between AgNPs could be fine-tuned by controlling experimental conditions, so as to form an optimized SERS hotspot. Moreover, RGO/[Ag(NH_3_)_2_] + (rGO/[Ag(NH_3_)_2_]+/Atp) paper tape modified with 4-thiosaminophenol (Atp) was prepared. It was used as SERS test paper and swab for the field determination of formaldehyde and acetaldehyde in wine, and the detection limits were 0.15 ng·L^−^^1^ and 1.3 ng·L^−^^1^, respectively. The SERS method, which is widely used for on-site analysis of volatile compounds in complex matrix samples, still needs further exploration to establish a more reliable sample pretreatment method with high extraction efficiency.

To ensure the quality and consistency of some alcoholic products, some edible additives, colorants, and necessary preservatives are added to improve the shelf life and taste. These additives include sulfite, sulfur dioxide, potassium sorbate, sodium citrate, acid orange, and bright blue [41]. In order to strictly control food safety and ensure consumer health, the monitoring of these ingredients is essential.

Sulfur compounds are widely used as preservatives and antioxidants in food and beverages, and they are commonly used in wine. Although sulfites are a common and accepted additive in wine, excessive consumption can affect consumer health. Sulfur dioxide levels in final wine products must be monitored and managed. By studying the interaction between AgNPs of different sizes and sulfur dioxide in aqueous alcohol solution, simulated wine, and wine samples, 4 nm AgNPs was selected for quantitative analysis of wine samples. The results showed that the limit of detection (LOD) of wines ranged from 0.6 mg/L to 9.6 mg/L, which was consistent with the International Organization of Grape and Wine Law (OIV-MA-AS323-04A) [152]. In 2021, Kong et al. verified the good performance of SERS for sulfite detection [153].

SERS has been used in combination with other technologies to determine the chemical composition of complex alcoholic beverage products, with high specificity for the detected substances. For example, thin-film microextraction (TFME), solid-phase micro-extraction (SPME), disperse magnetic solid-phase micro-extraction (DIS-MSPME) [154], headspace sampling (HS), and paper-based analytical devices (PAD) have been explored. In 2016, Chen et al. explored a simple and new method for on-site determination of sulfites in wine using a gas-diffusion microfluidic paper-based analytical device (µPAD) combined with SERS detection. The experiment showed that when the Raman displacement was 620 cm^−^^1^, SERS signal showed a good linear relationship with the concentration of SO_2_ in the range of 5–300 µg mL^−^^1^. The linear correlation coefficient was 0.995, and the detection limit was 2 µg mL^−^^1^ [155]. Based on the self-made Raman scanning system and SERS base, SC silver sol prepared by sodium citrate reduction method, the quantitative prediction model of potassium sorbate in *Osmanthus fragrans* flower wine was established. The model realized the quantitative prediction analysis of potassium sorbate, and it could be applied to the prediction of potassium sorbate in other fruit wines [156]. Xie et al. synthesized a new type of core-shell nanomaterial and detected acidic orange and bright blue in red wine through SERS. The core-shell nanomaterial was found to have a good SERS effect. The results showed that under the optimal experimental conditions (pH 6.02, mixing time 20 min), the detection limit of lime II was 1 μg/mL. The detection limit of brilliant blue was 0.5 μg/mL. The method was verified by HPLC, and the results showed that the determination of pigments in wine was effective [157].

### 4.3. Detection of Harmful Ingredients

Some illegal adulterants and additives are not easy to detect by ordinary detection methods, such as sildenafil, phthalic acid esters (PAEs), and flibanserin [115]. Some determination methods have high sensitivity, such as GC/MS, LC/MS, nuclear magnetic resonance (NMR) spectroscopy, etc., but their developments are limited by the complex and time-consuming sample pretreatment process, large instrument, and high cost, which are not suitable for on-site detection. Xiao et al. [158] used OTR 202 as a SERS-active substrate for the detection of sildenafil. The Raman enhancement factor (EF) of OTR 202 colloid was 1.84 × 10^7^, and the LOD of sildenafil in health wine and liquor was as low as 0.1 mg/L. Compared with the previous research with LODs of 1 [159], 1.63, and 2.20 mg/L [160], the LOD of sildenafil in this study was improved greatly. Lin et al. [161] also introduced Opto Trace Raman 202 (OTR 202) as an active colloidal surface enhanced Raman spectroscopy (SERS) for the rapid quantitative determination of Sildenafil (SD) in alcoholic beverages, and the detection limit was 0.1 mg/L. Thus, SERS can be used for the rapid and quantitative determination of sildenafil in complex alcoholic beverages. Butyl benzyl phthalate (BBP) and other PAE behave similarly to estrogen, and a high intake of PAE will lead to reduced sperm count in men and altered sexual organ development in children. BBP-type compounds are often added to liquor; therefore, a quick and simple detection method is urgently needed. Zhou et al. [162] synthesized Ag@Fe_3_O_4_@Ag/β-cyclodextrin (CD) nanoparticles and used them as SERS-active substrate. The results showed that SERS simplified sample processing, improved the detection limit of PAE, and determined BBP concentrations in real wine samples; the detection limit was as low as 14.9 nM. Li et al. [163] constructed β-cyclodextrin (β-Cd)-stabilized AuNPs (AuNPs@beta-CD) colloid, and the corresponding SERS signal was amplified and abundant hotspot were obtained. The minimum detection limit of BBP was 0.01 μM. Liu et al. combined liquid-liquid extraction and self-assembled AuNPs for the dual-analyte detection of BBP in the organic phase and edible pigment, sunset yellow, in the aqueous phase [164]. One of the studies applied a colorimetric SERS sensor to evaluate BBP in liquor and yellow rice wine [165]. Wu et al. combined solvent-driven self-assembled Au NPs with SERS to detect additives in food. Due to the uniform distribution and high-density hotspot, the assembled substrate was highly sensitive and showed excellent uniformity and reproducibility. The sensitive analysis of ciprofloxacin (CF), diethylhexyl phthalate (DEHP), tartrazine, and azodicarbonamide at 0.1 ppm concentrations was achieved by SERS detection. The AuNP array was an effective SERS substrate for food additives detection [165]. The rapid detection of banned additives, such as saccharin sodium sweetener, in liquor was achieved by SERS. The limit of detection concentration of saccharin sodium and sweetener in alcohol reached 1 mg, and the detection time was less than 10 min. The results show that SERS is a rapid detection platform [166].

Other toxic substances have also been found in alcoholic beverages, such as micro-and nanoplastics [167], SO_2_, ethyl carbamate (EC), and mycotoxins [168] (i.e., ochratoxin A (OTA) Aflatoxin B1) [169]. Ethyl carbamate (EC) [170] is a potentially toxic compound that is widely found in fermented foods and alcoholic beverages (wine, beer, spirits, and Chinese rice wine) and has carcinogenic potential. Some new technologies have been applied to detect EC in alcoholic products. For example, HPLC [171], solid-phase extraction, gas chromatography (GC)/mass spectroscopy (MS), HPLC with florescence detection, and LC-MS/MS [172]. These methods have high accuracy and selectivity, but the application of these technologies has some disadvantages, such as high cost, complexity, and high organic solvent consumption, which cannot meet the requirements of detection in some beverage industries. Raman spectroscopy, especially SERS, is of particular interest because of its simple operation, sensitive detection, and ability to analyze aqueous samples without pretreatment. In 2013, Yang et al. used SERS technology in combination with individual Au@Ag NPS for the analysis of three alcoholic beverages (obstler, a fruit wine made from apples and pears; vodka; and white rum). A single silver-coated gold nanoparticle colloid was used as the SERS amplifier, and in situ EC detection was quickly and effectively realized [173]. Qi et al. prepared a flower-shaped silver nanostructured substrate and silver nanocube substrate, and they were used in a SERS platform. The flower-like silver substrate proved to have better Raman enhancement for EC and was selected for further EC detection. The feasibility and reliability of the method were verified by testing real alcoholic beverages [174]. Wu et al. developed a simple and reliable method for the rapid extraction and sensitive detection of EC in rice wine and fruit brandy, based on the combination of molecular-imprinted polymer and SERS (MIP5-SERS) to form a novel nanobiosensor. The sensor could quickly and effectively detect EC in alcoholic beverages [175]. OchratoxinA (OTA) is a mycotoxin secreted by *Aspergillus* and *Penicillium*, and it is prevalent in alcoholic beverages. Song et al. developed an aptamer sensor for OTA using Fe_3_O_4_@Au magnetic nanoparticles (MGNPs) and Au@Ag nanoprobe modified with Raman reporting molecule, 5,5-dithio-bis-(2-nitrobenzoic acid) (DTNB). The Raman signal was significantly enhanced. When the OTA aptamer combined with OTA in the sample, the Raman signal decreased according to OTA concentration in the sample [169]. In combination with more efficient enrichment methods, the detection limit of the sensor can be further improved [176]. The continuous exploration and innovation of this technology also provides a more sensitive substrate or sensor for the detection of many different substances. Huo et al. [177] designed a smart porous Nu-901 film wrapped on mercapto magenta-modified Ag nanoparticles (TM-Ag@NU-901) as a color SERS sensor for the detection of sulfur dioxide (SO_2_) in a recent study. Combined with a portable Raman spectrometer, this method can be used for routine field detection of SO_2_ in alcoholic beverages quickly and efficiently, with the time and economic cost of a sample analysis being less than 2 min and 0.1 USD, respectively. The LOQs of TM-Ag@NU-901 film towards SO_2_ was 1 μM in wine samples, which is much lower than the acceptable limit of SO_2_ in wine (2.5 mM) set by the European Union.

SERS has been recognized by the field of harmful substance detection, including (1) Innovation on active substrates; (2) Combination with chemical analysis methods; (3) Combination with the sample pretreatment method; (4) Preparation of sensors. Through continuous exploration, the method has been verified in the specificity, sensitivity, non-destructive testing, and field testing of alcoholic beverages. The detection of harmful substances in alcoholic beverages using SERS in recent years has been summarized in Table 3.

### 4.4. Microbiological Detection

The existence of microorganisms in alcoholic beverages is very common. Brewing microorganisms are generally divided into three categories: alcoholic microorganisms (yeasts), saccharifying microorganisms (filamentous fungi), and bacteria. The alcoholic microbes include beer yeast, schizophytic yeast, and odoriferous yeast. Saccharifying microorganisms include *Aspergillus*, *Rhizopus*, and *Monascus*. Bacteria include lactobacillus, caproic acid bacteria, acetic acid bacteria, and butyrate bacteria. For example, *Brettanomyces/Dekkera* yeast appears in wine making and causes spoilage [178]. SERS has been widely applied in the detection of microorganisms, including fungi [179], bacteria [5], pathogen microorganisms [180], etc. Kashif et al. [38] combined SERS with PCA and PLS-DA to detect and distinguish bacteria in food processing with an accuracy of 99.5% and a sensitivity of 99.7%, which proved that SERS was an effective technology to identify and distinguish bacteria in food processing. *Brettanomyces bruxellensis* is an important spoilage yeast that causes “brucella odor” in wine. It is considered one of the most complex and controversial issues in aging red wine. SERS and localized surface plasmon resonance (LSPR) were used for the detection of target yeast ssDNA. Ionescu et al. designed a new gene sensor (Brett DNA) on a nanostructured superfine cover glass, and SERS was used for the identification of *Brettanomyces bruxellensis*. The detection limit was 0.1 ng/mu L (57.2 nM). The dynamic range was very wide and better than LSPR detection results. The gene senor was used to identify 14 kinds of yeast in the grape planting environment, and the feasibility of the deice was confirmed [181]. In 2013, Rodriguez et al. used Raman technology in combination with stoichiometric classification tools to isolate and identify three yeast species in finished wine, and the method achieved bacterial strain level classification with an overall accuracy of 81.8% [182].

## 5. Summary and Prospect

SERS has been widely used in the fields of food, biology, and medicine. This technology combines the specificity of molecular fingerprint identification with the potential of single molecule sensitivity. It is a versatile technique with great application prospects. This paper summarized SERS technology in alcoholic beverage analysis, and we concluded the innovation of SERS technology mainly includes the following aspects: (1) The innovation of the metal substrate, including the exploration and development of material shape and structure, greatly enhanced the Raman signal of the analyte; (2) Innovation of substrate materials, such as the discovery of graphene, silicon dioxide, and other materials, has the advantage of reducing costs and analysis time; (3) The effective composition of composite materials has been shown to enhance Raman spectral characteristics; (4) The combination of SERS with other technologies improves the sensitivity and specificity of detection; (5) Combined with theoretical analysis method and machine learning, the results of this technology can be more intuitive and clear. Alcoholic beverages are special products with a complex composition. Hence, it is necessary to develop a convenient, rapid, and sensitive detection technology that is resistant to interference by ethanol and water and realizes on-site detection. The use of SERS technology for the analysis of basic components, additives, beneficial components, toxic and harmful components, microorganisms, and metabolites in alcoholic beverages was summarized herein. The continuous innovation of SERS technology can further enhance its sensitivity, convenience, and stability in the detection of alcoholic beverages.

Thus far, the application of SERS technology in the detection of alcoholic beverages is still in the stage of continuous experimentation and exploration. Due to the characteristics of alcoholic beverages, including trace number of components, complex type, and high content of water and ethanol, it is necessary to continuously explore the appropriate enhanced substrate to promote the ability of SERS field detection. In addition, hotspot signals, based on incident light and vibration of molecular bonds, can be generated to enhance SERS detection, and this strategy can be applied to the detection of trace analytes in complex samples. We have reasons to believe that future developments of SERS detection may focus on tailoring the parameters of Raman instruments, developing various active substrates with high sensitivity, good stability, and excellent reproducibility, and combining some pretreatment methods and analysis techniques. Moreover, a low-cost, small Raman spectrum analyzer with simple operation, rapidity, sensitivity, stability, and portability will also be the focus of instrument research and development.

In short, taking the detection of complex alcoholic beverages as an example, this review systematically summarizes the development and detection application of SERS technology. This review provides data reference for the further development of SERS technology and its application in other food industries.

## Figures and Tables

**Table 1:** Composition proportion of alcoholic beverages and its impact on alcoholic beverages.

Alcoholic Beverages	Composition	Content (%)	Impact
**Main ingredients**	Water and alcohol	85–95	An essential ingredient in alcoholic beverages
**Basic ingredients**	Higher alcohols, lipids, aldehydes and ketones, furans, aromatic compounds, pyrazines, acids, etc.	3–9	Various taste
**Active ingredients**	Pyrazines, terpenes, polyphenols, flavonoids, amino acids, etc.	1–3	(1) Characteristics of different liquor components; (2) Endow alcoholic drinks with healthy elements; (3) Beneficial to human health, etc.
**Toxic and harmful ingredient**	Food additives, adulterants, methanol, formaldehyde, cyanide, metal ions, pesticide residues, mycotoxins, etc.	0.5–1.5	(1) Increase the shelf life; (2) Improve the taste or color; (3) Affect the quality of wine, etc.
**Microorganism**	*Saccharomyces cerevisiae*, beneficial microorganisms, etc.	1–1.5	(1) Fermented alcoholic beverages are essential; (2) The source of aroma and taste substances; (3) Affect the quality of wine, etc.

**Table 2:** Application of SERS in the identification and characterization of alcoholic beverages in recent years.

Types of Alcoholic Beverages	SERS and Related Parameters Adjustment	Conclusion
white wine [145]	Unlabeled SERS spectra were combined with multivariate data analysis (principal component analysis SIMCA prediction model), with colloidal dispersions of Ag nanoparticles as substrates Instrument and parameters: Portable I-Raman Plus 785S Spectrometer (equipped with a 785 nm emitting 300 mW diode laser and a spectrometer with a high quantum efficiency CCD array (cooled at 2 °C)) with laser power of 320 mW. The total collection time was 10 s (5 s for two accumulative times)	(1) Accurate prediction of which wine producer it was, with a sensitivity of 91% and a specificity of 95% (2) Excellent efficiency (87.93%) in distinguishing specific wines or specific producers (3) The main spectral differences were caused by adenine, carboxylic acid, and glutathione
white wine [59]	Silver Nanostars (AgNSs), SERS combined with data analysis software (SPSS program, stepwise linear discriminant analysis (SLDA)) Instrument and parameter: compact DeltaNU532 Raman system equipped with a frequency doubled Nd: YAG laser operating at 532 nm line (output power 100 mW). Five seconds of five accumulations have been set for each spectral acquisition in the 200–3350 cm^−1^ spectral range.	(1) Successfully distinguished three different white wines (2) The method is proved to be simple, fast, and cheap
wine [61]	FT-Raman and chemometrics, Silver Nanoparticles (AgNPs) Instrument and parameter: compact DeltaNU532 Raman system equipped with a frequency doubled Nd: YAG laser operating at 532 nm line (output power 100 mW). Five seconds of five accumulations have been set for each spectral acquisition in the 200–3350 cm^−1^ spectral range.	(1) Some wines have a geographical differentiation rate of more than 90%, while Romania’s 372 varieties have a geographical differentiation rate of 83.3%.
wine [147]	Silver Nanoparticles (AgNPs) and FT-Raman spectra (Bruker Equinox 55 FT-IR spectrometer with an integrated FRA 106S Raman module) Instrument and parameter: Detection was achieved with a liquid nitrogen-cooled Ge detector. Spectral resolution was 4 cm^−1^. Laser power was set to 350 mW and 500 scans were co-added.	(1) Ag NPs had a strong interaction with wine components, and showed induced aggregation. The main SERS signal characteristics for anthocyanins were under 532 nm excitation.
Red wine [148]	Method: direct analysis of red wine by Raman spectroscopy; mixed with silver nanoparticles, known as AgNPs; a reproducible SERS substrate, AgNPs mirror; SERS was combined with solvent extraction	(1) AgNPs images can reduce fluorescence (2) An innovative method based on extraction successfully suppressed the dominance of adenine in SERS spectroscopy
	Instrument and parameter: ChemLogix EZRaman-I Series High Performance Portable Raman Analyzer with a 785 nm laser source, under following conditions: 170 mW laser power, 5 times integration, and 2 s exposure time.	(3) These chemicals form a barcode that could potentially be used to determine the classification and authenticity of a wine

**Table 3:** Summary of the detection of harmful substances in alcoholic beverages using SERS in recent years.

Composition	SERS Platform and Related Parameters	Limit of Detection (LOD)	Conclusion
**Sulfur dioxide (wine)**	(1) Active substrate: silver nanoparticle (AuNPs) Instrument and parameter: Thin-film microextraction (TFME) + SERS (2) Active substrate: The preferred combination of silver nanoparticles (AgNPs) with sulfur-containing substances (ratio 1:1) Instrument and parameter: Thermo Fisher Scientific DXRxi Raman Spectrometer (Waltham, USA), with a 532 nm excitation laser source and a charge coupled device (CCD) detector Spectra were collected with a 10-mW laser power and a 50 μm rectangular aperture in the spectral range of 50 cm^−1^ to 3400 cm^−1^. Each spectrum was collected for 1 s and exposed 50 times. Repeat each measurement at least three times	(1) SERS signal intensity at 600 cm^−1^ had a good linear relationship with SO_2_ concentration in the range of 1–200 ug/mL, and the linear correlation coefficient was 99.2%. The detection limit of SO_2_ was 0.1 ug/mL [119].	Both studies showed that SERS could be a simple, rapid, and selective method for the determination of SO_2_ content in wine. In the first study, the detection limit of SO_2_ was reduced by combining the pretreatment method. It shows that SERS technology combined with the pretreatment method is more advantageous.
		(2) Regulation (EU) sets the legal limit for the amount of SO_2_ in red wine at 150 mg/L and white wine at 200 mg/L.& The detection limit (LOD) was 0.6 mg/L to 9.6 mg/L [152].	
**Sulfur dioxid(SO_2_),** & **sulfite** [154] **(wine)**	Active substrate: combined with surface-enhanced Raman Spectroscopy (SERS), AuNPs dispersed on the substrate of sea urchin-like ZnO nanowire. Instrument and parameter: Gas-diffusion microfluidic paper-based analytical device (PAD) + SERS. A portable Raman instrument (I-Raman, B&W Tek Inc., USA) with a microscope (20 objective) is used for acquisition. The laser excitation wavelength is 785 nm.	The SERS signal displacement was 620 cm^−1^, and the SO_2_ concentration showed a good linear relationship in the range of 5–300 μg mL^−1^. The linear correlation coefficient was 0.995 and the detection limit for sulfite was found to be 2 μg mL^−1^.	(1) This method is endowed with portability, minimal reagent consumption, and operational simplicity; (2) This method has good selectivity in sulfite analysis of wine samples.
**Sulfite** [155] **(liquor)**	Method: A novel paper-based analysis device for gas diffusion microfluidics (PAD) combined with surface-enhanced Raman spectroscopy (SERS) Active substrate: Sea urchin-like nano ZnO-paper Instrument and parameter: Portable Raman instrument with microscope (20 objective) (I-Raman, B&W Tek Inc., USA). The laser excitation wavelength is 785 nm.	The SERS signal displacement was 620 cm^−1^, and the SO_2_ concentration showed a good linear relationship in the range of 5–300 g mL^−1^. The linear correlation coefficient was 0.995, and the detection limit was 2 g mL^−1^.	(1) This method would permit a fast, disposable, and economical routine on-site monitoring of sulfite.
**Sildenafil** [161] **(cocktail)**	Active substrate: Opto Trace Raman 202 (OTR 202) colloidal activity Instrument and parameter: (1) RmTracer-200-HS portable Raman spectrometer combined with a 785 nm excitation wavelength diode-stabilized stimulator (Opto Trace Technologies, Inc., Mountain View, CA, USA); (2) FEI Tecnai G2 F20 S-TWIN transmission electron microscope. (3) Density functional theory (DFT). A 785 nm excitation wavelength, a power of 200 mw, a scanning range of 200–3300 cm^−1^, an optical resolution of 2 cm^−1^, an integration time of 10 s, and an average spectral value of three times.	The detection limit (LOD) was as low as 0.1 mg/L	(1) SERS technology can quickly and quantitatively determine SD in cocktails; (2) It is conducive to providing a fast and accurate scheme for the detection of SD in alcoholic drinks.
**Sildenafil** [158] **(white wine, wine, health wine, etc.)**	Active substrate: Opto Trace Raman 202 (OTR 202) active colloids Instrument and parameter: (1) RmTracer-200-HS portable Raman spectrometer combined with a 785 nm excitation wavelength diode-stabilized stimulator (Opto Trace Technologies, Inc., Mountain View, CA, USA); (2) FEI Tecnai G2 F20 S-TWIN transmission electron microscope. a 785 nm excitation wavelength, a power of 200 mw, a scanning range of 200–3300 cm^−1^, an optical resolution of 2 cm^−1^, an integration time of 10 s, and an average spectral value of three times.	There was a good linear relationship between the intensity of Raman peak and the concentration of sildenafil in health wine and liquor. The Raman enhancement factor (EF) of OTR 202 colloids reached 1.84 × 10^7^ and the limits of detection (LODs) of sildenafil in health wine and liquor were found to be as low as 0.1 mg/L.	(1) The Raman EF of OTR 202 colloids could reach 1.84 × 10^7^ (2) The proposed method showed good performance
**Flibanserin** [115] **(white wine, beer, and wine)**	Active substrate: AgNPs Instrument and parameter: SERS technology was combined with a machine learning algorithm (PCA, SVM). Portable Raman spectrometer BWS415-785H (B&W Tek, Inc.) with a wavelength of 785 nm. The spectrometer provided a 175–2000 cm^−1^ range of spectral measurement and the spectral resolution of the spectrometer was better than 3 cm^−1^. The output power was fixed at 150 mW and the integration time was 20 s.	The limit of detection of 1 g/mL for Flibanserin in liquor	(1) The results showed that this method can quickly and accurately detect Flibanserin in different wine solutions
**Ethyl carbamate** [173] **(vodka, obstler, and white rum)**	Active substrate: Individual silver-coated gold nanoparticle colloids (Au@Ag NPs) Instrument and parameter: Raman microscope (LabRAM HR, HORIBA Jobin Yvon, Germany), using a × 10 objectives. The 633-nm line of HE-NE laser was used as the excitation source. The laser power at the sample site was set to 14 mW. The spectral range is from 200 to 2000 cm^−1^ and the recorded resolution is 1 cm^−1^.	Control Board of Ontario (Canada) established the upper limits for EC in alcoholic beverages as ranging between 30 and 400 μg/L. The detection limits were 9.0 × 10^−9^ M (0.8 μg·L^−1^), 1.3 × 10^−7^ M (11.6 μg·L^−1^) and 7.8 × 10^−8^ M (6.9 μg·L^−1^), respectively, and the SNR was 3.	(1) The characteristic band at 1003 cm^−1^ was the strongest peak with the best reproducibility in SERS spectrum, which could be used for quantitative evaluation of ethyl carbamate
**Sulfur dioxide (SO_2_)** [174] **(wine)**	Active substrate: Porous NU-901 wrapping on thiol-magenta modified Ag nanoparticles (TM-Ag@NU-901) Instrument and parameter: Portable Raman spectrometers.	The detection limit is 1 μM, far lower than the acceptable limit of SO_2_ in wine (2.5 mm) stipulated by the European Union.	(1) It has the advantages of visual visualization, specificity, sensitivity, low cost, and time
**Ochratoxin A(OTA)** **(wine)**	Active substrate: AgNPs Instrument and parameter: Solvent-mediated liquid-liquid extraction (LLE) + Thermal science DXRxi Raman spectroscopic microscopy 20× objective, 780 nm excitation wavelength, 5 mW laser power, 50 μm slit aperture and 0.01 s collection time for an area of 2.8 mm × 2.8 mm. Active substrate: Silver (Ag)-capped silicon nanopillars substrate Method: SERS + high throughput supported liquid membrane (SLM) extraction. Instrument and parameter: DXRxi Raman Imaging Microscope, 10× objective lens, 50 μm slit, and an estimated laser spot of 3.6 μm diameter. All the spectra were collected three times for 0.05 s in each spot.	There is a good linear correlation between Raman intensity and OTA concentration; the correlation coefficient R = 0.9938, which is within the range of 0.01–1 ppm [63].	Both methods can detect Ochratoxin A in wine quickly and without damage. The detection limit of the first method was lower, up to 0.01 PPM.
		The detection limit is 115 PPB [176].	
**Ethyl carbamate** [175] **(wine, yellow wine and fruit brandy)**	Active substrate: Silver dendrite nanostructure Instrument and parameter: Molecularly imprinted polymers and SERS (MIPs SERS) (with an air-cooled He–Ne laser for 785 nm excitation, a motorized microscope, and a CCD array detector with 1024 × 256 pixels was used to record the SERS spectra).	~	(1) The MIPs-SPE can successfully separate EC and other components from wine samples. (2) SERS spectroscopic collection can be completed within 10 s, greatly shortening the detection time.
**Histamine** [62] **(wine)**	Active substrate: Silver nanoparticles (AgNPs)-cellulose hybrid substrate Instrument and parameter: Thermogravimetric analysis (TGA), scanning electron microscopy (SEM), and Raman spectroscopy (Raman).	The high sensitivity of the promoted SERS phenomenon allows detecting histamine 10^−12^ M concentrate	The cellulose device is sensitive to histamine detection
**Butyl Benzyl Phthalate** [163] **(liquor,** **wine)**	Active substrate: AuNPs (AuNPs@β-CD) Instrument and parameter: The LAB-RAM HR800 spectrometer is equipped with a 633 nm laser excitation source and a laser power of 0.9 mW.	Detection limit as low as 14.9 nM	(1) A novel and effective solvent discoloration and SERS sensor system has opened up a new way.
**Phthalate esters** [162] **(PAEs)** **(liquor)**	Active substrate: Ag@Fe_3_O_4_@Ag/β-cyclodextrin (CD) nanoparticles Instrument and parameter: LabRAM HR800 confocal microscope and a 1-mW, 633-nm He-Ne laser (with a LWD 50/0.5 NA objective lens, and the laser spots had a diameter of about 2.0 μm; the acquisition time was 1 s for MG and 5 s for the other analytes.)	The detection limit was 1 ppb	(1) This substrate could detect that the level of BBP in liquor was reduced to 1.3 mg/kg, which was low enough to detect BBP in liquor samples
**Formaldehyde(FA) and acetaldehyde(AA)** [151]	Active substrate: Silver nanoparticle (AgNPs), RGO/[Ag(NH3)2] + (rGO/[Ag(NH3)2] + /Atp) paper Instrument and parameter: Portable Raman spectrometers.	The detection limits were 0.15 and 1.3 ng·L^−1^	(1) The establishment of sample pretreatment method is a potential way to improve SERS detection of samples
**Acid orange II and brilliant blue** [157] **(Red wine)**	Active substrate: Fe_3_O_4_ @Au nanoshells Instrument and parameter: Portable RamTracer-200-HS Raman spectrometer (An excitation light source of 785 nm for an average scan time of 20 s, the number of integrals was two times, a scanning power of 300 mW, a sweep range of 100–3300 cm^−1^).	The detection limit of lime II was 1 μg/mL. The detection limit of brilliant blue was 0.5 μg/mL	(1) The method was verified by HPLC, and the results showed that the determination of pigments in wine was effective

## Data Availability

No new data were created or analyzed in this study. Data sharing is not applicable to this article.
